# Bioinformatic Approaches to Validation and Functional Analysis of 3D Lung Cancer Models

**DOI:** 10.3390/cancers13040701

**Published:** 2021-02-09

**Authors:** P. Jonathan Li, Jeroen P. Roose, David M. Jablons, Johannes R. Kratz

**Affiliations:** 1 Department of Surgery, University of California, San Francisco, CA 94143, USA; po-yi.li@ucsf.edu (P.J.L.); david.jablons@ucsf.edu (D.M.J.); 2 Department of Anatomy, University of California, San Francisco, CA 94143, USA; jeroen.roose@ucsf.edu

**Keywords:** lung cancer, 3D cultures, organoids, spheroids, xenografts, bioinformatics

## Abstract

**Simple Summary:**

Lung cancer remains a major cause of mortality worldwide. Treatment options for lung cancer have remained relatively unchanged despite a significant need, and unrepresentative preclinical cancer models contribute to stifled therapeutic development. 3D models of cancer, including xenografts, spheroids, and organoids, have the potential to improve cancer research and drug development because they are more representative of cancer biology and its diverse pathophysiology.

**Abstract:**

3D models of cancer have the potential to improve basic, translational, and clinical studies. Patient-derived xenografts, spheroids, and organoids are broad categories of 3D models of cancer, and to date, these 3D models of cancer have been established for a variety of cancer types. In lung cancer, for example, 3D models offer a promising new avenue to gain novel insights into lung tumor biology and improve outcomes for patients afflicted with the number one cancer killer worldwide. However, the adoption and utility of these 3D models of cancer vary, and demonstrating the fidelity of these models is a critical first step before seeking meaningful applications. Here, we review use cases of current 3D lung cancer models and bioinformatic approaches to assessing model fidelity. Bioinformatics approaches play a key role in both validating 3D lung cancer models and high dimensional functional analyses to support downstream applications.

## 1. Introduction

In the past decade, promising progress in our understanding and treatment of cancer has been made. These advancements have helped contribute to increased declines in death rates for some cancer types [1]. Continued improvements in cancer care and the realization of personalized cancer medicine will require better characterization and modeling of cancer. Increasingly widespread application of high dimensional analysis tools, such as single cell sequencing, to cancer research has already led to studies of cancer evolution, cancer immunobiology, and the tumor microenvironment at a greater resolution than ever before [2,3,4]. These studies have both underscored the diverse pathophysiology of cancer and uncovered potential novel therapeutic targets. Validating and translating these nuanced observations to improved patient care now demand cancer models that more fully recapitulate the complex and heterogenous manifestations of cancer.

Because of their significant potential to improve drug discovery pipelines, preclinical cancer models are an active area of research and development. Traditional cancer drug discovery involves screening libraries of anticancer compounds in a 2D model of cancer, such as a cancer cell line. While 2D models are advantageous for their ease of use, they do not accurately recapitulate the in vivo tumor microenvironment, and compounds identified using these models often do not make it into the clinic [5,6]. Thus, dependence on poor preclinical models is one contributing factor to the current low clinical approval rate for cancer therapeutics [7,8].

Beyond strategies targeting cancer cells, advancements in immunology and the success of immunotherapy in treating certain cancers have highlighted the critical role our immune system plays in shaping tumor evolution and controlling tumor growth [9,10]. The promise of immunotherapy has catalyzed a deeper dissection of how the tumor and its microenvironment modulate immune cell function, particularly since only a subset of patients currently experiences durable progression-free survival with immunotherapy [4,11,12]. Immuno-oncology drug development is now a rapidly growing sector, and the complex nature of the immune system further demands improved preclinical models to facilitate the development of next generation of cancer therapies [13,14].

## 2. 3D Preclinical Models for Cancer Research

**Figure 1:**
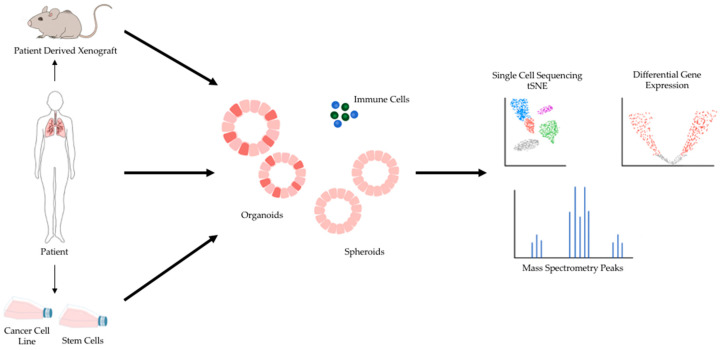
3D models of cancer can be generated from a variety of sources, including mouse models of cancer, patient specimens, and pre-existing cancer cell lines. Additionally, patient specimens can be used to generate xenograft models in mice, and human stem cells can be reprogrammed into desired cell types and subsequently cultured in 3D. 3D models of cancer can comprise just cancer cells, as most currently do, or include additional components such as immune cells. A variety of sequencing modalities and mass spectrometry are among the many downstream analytical pipelines 3D models of cancer can be processed through.

The shortcomings of 2D models of cancer have directed increasing attention towards 3D models of cancer. Studies comparing 3D cancer models with their 2D counterpart have demonstrated differences in gene expression and signaling pathway activity and highlighted the potential for 3D models to enable more accurate drug screens [15,16,17]. In general, 3D models are considered a favorable alternative because they preserve natural cell shape, cell growth, nutrient gradients, cell-to-cell communication, and gene/protein expression levels better than 2D models [6]. Several 3D cell culture models have been developed over the years, and 3D models of cancer are being increasingly adopted for research and therapeutic development [5]. Major types of 3D models of cancer include patient-derived xenografts (PDX), spheroids, and organoids (Figure 1).

PDX models involve grafting human tumor tissue onto humanized or immunodeficient mice. In general, PDX models accurately model host tumor characteristics, such as histopathology, gene expression profile, and genomic alterations, but their primary limitations include low establishment rates and long propagation times [18,19]. The lengthy and costly process of establishing PDX models may limit their use for high-throughput screens [19,20]. Additionally, studies using PDX models have noted the accumulation of PDX-unique single nucleotide variants when compared back to the donor tumor, although it is unclear whether this is a xenograft-mediated selection process or whether it represents natural tumor evolution [18]. The lack of a functional immune system of most PDX host mice is also a significant limitation for developing novel immune-based therapies.

Spheroids are in vitro cultures of 3D homogenous or heterogenous cell aggregates. This culture technique started in the late 1980s, facilitated by the advent of Matrigel, with the primary goal of replicating tissue-specific morphology and protein production [21]. Multiple methods have since been developed to propagate spheroids, with the main difference being whether the spheroids are formed with materials that mimic the extracellular matrix (scaffold-based) or without (scaffold-free) [22]. In current cancer research, tumor spheroids consisting of cancer cells, most commonly from a cancer cell line, with or without the addition of cells such as fibroblasts and endothelial cells, are used to more accurately model solid tumor composition. Tumor spheroids have been shown to model the metabolic gradient of solid tumors more accurately than 2D models and have gene expression and drug susceptibility profiles concordant with in vivo tumors from a variety of solid cancers, including melanoma and colorectal cancer [22,23].

Organoids are also 3D in vitro cultures but are instead composed of a heterogenous cell population including stem cells from primary tissue that grow and self-organize into structures resembling their organ derivative [24,25]. Culture conditions that support the retention of stem cells and, subsequently, the ability of organoids to stably self-renew over sustained culture periods and self-organize are the primary factors that differentiate organoids from spheroids. These features make organoids promising models for studying development, tissue homeostasis, and rare diseases in addition to cancer [24]. In the context of cancer research, organoids have been successfully cultured from multiple normal and cancer tissue types including lung, breast, pancreas, and colon [23,26].

Other noTable 3D preclinical models for cancer research include patient-derived explants (PDE)—ex vivo cultures of freshly resected tissue fragment. PDEs are potentially advantageous because they preserve original tissue architecture, enabling more accurate spatial characterization of biomarkers in the tumor microenvironment, and have been shown to also replicate patient drug responses well [27]. However, PDEs are highly dependent on the quality of surgical resection, are not as amenable to high-throughput studies, and have yet to achieve as widespread adoption as PDX, spheroids, and organoids in translational cancer studies [27].

Overall, these 3D models offer unique advantages over 2D models and will enable improved preclinical cancer studies. The current utility and adoption of these models varies in the study of different cancer types, but they have especially promising applications in cancers with poor prognoses, such as pancreatic ductal adenocarcinoma and metastatic cancers [28,29]. Additionally, while most of the models discussed still exclude important elements of the tumor microenvironment such as stroma and immune cells, next generation 3D models, including new organoid culture techniques that incorporate immune cells, are beginning to enable studies of cancer–immune cell interactions [30,31,32,33,34,35,36,37]. Coupled with high dimensional genomics and proteomic profiling, these advancements further extend the utility of 3D preclinical cancer models, with implications for immunotherapy response prediction and immune-based therapy development for solid cancers.

## 3. Lethality of Lung Cancer and the Need for Improved Models

Lung cancer outcomes could greatly benefit from improved preclinical models and therapeutic development. Each year, lung cancer is diagnosed in over 1.8 million people worldwide and is the cause of 1.6 million deaths [38]. For the year of 2020, the America Cancer Society reported 228,820 new cases of lung cancer and 135,720 deaths due to lung cancer in the United States alone [1]. Early detection of lung cancer increases the number of patients who undergo potentially curative surgery, but even after complete surgical resection, early-stage cancers recur up to 30–50% of the time within 5 years [39]. Despite this high recurrence rate, the current standard of care after resection of most stage I lung cancers is “observation”, a treatment paradigm which has not changed over the past several decades. Thus, there is a significant need to better understand lung cancer biology and create novel treatment options.

In this review, we will focus on the fidelity of 3D lung cancer models and how 3D lung cancer models are yielding new insight into disease and treatment options. Importantly, we will highlight the bioinformatics tools that have enabled the validation and functional characterization of these models (Table 1).

## 4. Sequencing Validation of Patient-Derived 3D Lung Cancer Models for Drug Efficacy Screens

To improve the discovery process of anticancer compounds and to accelerate the validation of novel treatment strategies, preclinical 3D models of lung cancer need to accurately capture the architectural, cellular, and genomic composition of patient tumors. Assessing these characteristics is a critical first step, especially since there are notable variations in 3D culture methodologies across the field. For example, lung organoid culture media composition can vary greatly [40,41,42]. In addition, differences in primary tissue acquisition (biopsy vs. surgical resection) and downstream tissue processing for lung cancer organoid establishment can result in varying purities of the established cancer organoids [43]. Together, these factors may serve as unaccounted selective forces that skew organoid phenotypes. Thus, it is important to validate how well an organoid model actually recapitulates patient phenotypes when establishing or modifying organoid culture techniques.

Histological comparison of the 3D model of interest and its tissue derivative offers a quick qualitative way to assess the fidelity of architectural and cellular composition. To assess the fidelity of genomic composition, sequencing methodologies, such as whole exome sequencing and RNA sequencing, can quantify how well organoids capture the patient’s mutational profile and preserve the unique gene expression profile of each cancer. As cancer is considered a genetic disease, these validation steps are necessary prior to proceeding to functional assays, such as predicting organoid response to established treatment regimens and potential novel combination therapies.

Several recent studies demonstrating the efficacy of 3D lung cancer models in predicting treatment response utilize this workflow [41,44,45,46]. A comprehensive study by Izumchenko et al. examined the clinical utility of PDX models for a variety of cancer types, including lung cancer [44]. Using whole exome sequencing, the study compared mutation rates and the spectrum of base pair substitutions of the established PDX models to corresponding TCGA or host tumor data. When compared to TCGA data, the authors noted similar PDX mutation rates and spectrum of base pair substitutions for different cancer types, although for non-small cell lung cancer (NSCLC), there were differences in the frequency of smoking related C > A transversions. Promisingly, for NSCLC, there was strong concordance of base pair substitution distribution between PDX and matched host tumor tissue, although the sample size for this was small (*n* = 4). Additionally, when patient treatment regimens were simulated in their corresponding PDX models, the authors noted high concordance in response and drug efficacy. When determining the diagnostic efficacy of PDX models, the authors found an overall sensitivity of 96% and a specificity of 70% across all cancer types evaluated (breast, colorectal, ovarian, lung, gastroesophageal, pancreatic, sarcoma). Based on these findings, PDX models demonstrate utility in validating patient treatment regimens and screening for novel drug combination and small molecules. Despite the strong diagnostic performance, the authors did note that for NSCLC, the establishment rate was 50% and that, in general, PDX establishment took roughly 16 weeks. While PDX are promising in terms of model fidelity, the varying establishment success and long establishment times may have implications for the practical application of PDX models in patient treatment decision making.

A series of papers conducted similar validation studies on lung cancer patient-derived organoids (PDOs). One study by Chen et al. analyzed a small cohort of paired NSCLC PDOs and tumor resections [45]. Based on whole exome sequencing analysis, the study found concordance of somatic mutations detected in organoids and corresponding tissue ranging from 50 to 80% as well as a relatively well conserved distribution of base pair substitutions. A separate study by Shi et al. focused on how well NSCLC organoids preserved the genomic profiles of their parental tumors compared to PDX models over long-term culture (defined here as >3 months or 10 passages) [46]. Using whole exome sequencing and RNA sequencing, the study confirmed that organoids cultured long-term were able to preserve both the mutational profile and gene expression profile. Concordant with previous preclinical studies, the study also found that both NSCLC PDOs and PDXs with *KRAS* mutations were selectively susceptible to mitogen-activated protein kinase kinase (MEK) inhibitors. Additionally, the study successfully trialed a novel combination therapy for lung squamous cell carcinoma (LUSC) with *FGFR1* amplification refractory to fibroblast growth factor receptor (FGFR) inhibitor monotherapy that consisted of an FGFR and MEK inhibitor using their PDX model.

One final study by Kim et al. compared the genomic profiles of long-term cultured organoids (defined here as >10 passages) to short-term cultured organoids [41]. Using whole exome sequencing and variant allele frequency analysis, this study confirmed that their culture method yielded organoids that preserved the genomic profile of the original tumor tissue and that long-term culturing did not result in deviation from the original genomic profile. Using these validated organoids, the study proceeded with drug response assays and uncovered two potential areas of additional exploration. One pair of organoids in this study had different breast cancer gene (BRCA) mutations that resulted in differential sensitivity to Olaparib. Follow up protein function prediction analysis suggested that one BRCA mutation was non-pathogenic, potentially explaining the different sensitivity to BRCA targeting drugs. Similarly, another pair of organoids shared the same epidermal growth factor receptor (EGFR) mutation but had differential response to Erlotinib. Follow up studies identified that one had an amplification of the *MET* gene and targeting with Crizotinib instead suppressed organoid viability.

Together, these studies demonstrate the fidelity and promising utility of PDX and PDO models in advancing lung cancer treatment. The combined use of whole exome sequencing, variant allele frequency, and RNA sequencing validated the genetic composition of these patient-derived models. Once validated, these models facilitated “personalized” studies of small molecule efficacy and discovery of more effective indications and combination therapy strategies.

## 5. 3D Models of Cancer, Single Cell Profiling, and Genomic Screens as Scalable Discovery Platforms

While patient tumor tissue and patient-derived 3D models of cancer may offer the best snapshot of disease states, their limited quantities can be a bottleneck in mechanistic studies and screens that demand greater tissue/cell input [43]. Thus, assessing the fidelity of 3D models of cancer derived from cancer cell lines, mouse models of cancer, and/or induced pluripotent stem cells in addition to those directly derived from patient tumor tissue is also important. Combined with single cell sequencing, these additional models serve as scalable discovery platforms.

A recent study by Dost et al. used single cell sequencing and a variety of non-patient-derived lung cancer organoid models to characterize the early transcriptional changes that occur following mutant KRAS activation, something of particular interest due to the prevalence of KRAS mutations and the difficulty targeting the KRAS pathway in epithelial cancers [47]. Using single cell RNA sequencing of transformed lung tissue derived from a genetically engineered mouse model of lung adenocarcinoma, the study initially identified alveolar type 2 (AT2) cells, epithelial cells in the lung responsible for secreting surfactant, as a cell population of interest in KRAS-driven lung adenocarcinomas. Next, the study formed organoids using untransformed AT2 cells isolated from the genetically engineered mouse model. Upon induction of mutant KRAS expression, transformed AT2 organoids histologically resembled their in vivo counterpart and formed tumors upon transplantation into mice. RNA sequencing of untransformed and transformed AT2 organoids followed by differential gene expression analysis found that transformed AT2 organoids had decreased expression of AT2 differentiation markers and increased expression of developmental lung markers. To see if these findings were relevant in humans, the study induced similar KRAS transformation in lung organoids composed of engineered AT2 cells derived from human induced pluripotent stem cells and observed similar de-differentiation of AT2 cells. Finally, the study completed single cell sequencing of tumor and normal lung tissue from two Stage 1A lung adenocarcinoma patients with KRAS activating mutations and validated the organoid findings that AT2 cells de-differentiate in early-stage lung adenocarcinoma. Although this study did not explicitly identify novel targets for cancer treatment, it did highlight the utility of multiple different lung cancer organoid models in elucidating early-stage lung cancer progression.

In another recent study, Han et al. utilized a lung cancer adenocarcinoma cell line and genome-wide screen to identify cancer-dependent genes that could serve as potential new targets [48]. First, the authors compared the genes that promoted or inhibited cell growth in 2D monolayer cultures of the H23 adenocarcinoma cell line versus those in 3D scaffold-free spheroid H23 cultures. The spheroid gene hit list was more representative of genes frequently mutated in lung adenocarcinoma and squamous cell carcinoma. Repeat screens in H1975 and H2009, both adenocarcinoma cell lines, confirmed that 3D spheroids were better platforms for studying cancer pathways and driver mutations than their 2D counterpart. Using DepMap, the study then grouped genes identified from the H23 screen based on function and identified the CPD gene module, among others, as a potential novel therapeutic target. To further explore this, the study used PRECOG, a reference database consisting of genomic profiles and cancer outcomes, to determine that high expression of *CPD* and its downstream genes was correlated with poor lung adenocarcinoma prognosis. Follow-up mechanistic studies further strengthened CPD as a prognostic marker and therapeutic target candidate for a particular subtype of lung adenocarcinoma. This study represents a powerful discovery pipeline and highlights the value of 3D cancer models for identifying the genetic bases of cancer. It also demonstrated the value of publicly available reference databases and bioinformatic tools that streamline hypothesis generation and identification of biologically relevant genes to use as prognostic markers and targets.

Taken together, these two recent studies highlight the utility of non-patient-derived 3D models of cancer, ranging from murine and human iPSC-derived lung organoids to human cancer cell line spheroids, in the study of lung cancer initiation as well as the identification of novel targetable pathways for therapy. In both studies, reference databases (PRECOG and TCGA) and bioinformatics tools (single cell sequencing analysis and pathway enrichment analysis) facilitated hypothesis generation, validation of the 3D models of cancer used, and the extraction of novel insights about lung cancer biology.

## 6. Single-Cell Profiling of Tumor-Microenvironments in 3D Lung Cancer Models

As described above, cancer immunology is a growing area of research interest and therapeutic development. Targeting inhibitory molecules, individually or in combination, has demonstrated success in solid tumors, such as melanoma, lung cancer, and prostate cancer, but the majority of patients still remain refractory to immunotherapy [49]. Novel immune cell-based therapies, such as CAR T Cells, are an up and coming therapeutic class but the majority have efficacy in “liquid” tumors [50].

With rapid discovery platforms such as CRISPR and pooled knock out and pooled knock-in methodologies being established, we are now able to uncover potential new therapeutic targets and engineer novel immune cell therapies at an unprecedented pace [51,52]. Similar to small molecule screens, these rapid genomic screening methodologies will require biologically accurate contexts to effectively and efficiently identify therapeutic strategies. As discussed above, 3D lung cancer models have the potential to become scalable discovery platforms for verifying cancer drug indications, uncovering new potential combination therapies, and identifying new pathways to target in cancer cells. In addition to cancer cell intrinsic factors, the tumor microenvironment becomes particularly important to consider during the development of immune-based therapeutics. For example, hypoxic cores, inhibitory soluble factors, and chemokine gradients may play important roles when designing and testing novel cell-based therapies or immune modulating substances. Immune repertoire and cell state are other important points of consideration for immune-based therapies. Existing checkpoint blockade therapies depend on the patient’s existing immune repertoire covering tumor antigens and those immune cells being in a state that can proliferate and eliminate transformed/cancer cells. On the other hand, engineered cell therapies can artificially expand the preexisting immune repertoire or diversify the immune repertoire. Cell therapies can also be engineered to be stable in a particular cell state by engineering switch receptors that rewire cell signals or transcription factors that maintain particular gene circuits [53]. Subsequently, 3D lung cancer models that recapitulate the complexity of the tumor microenvironment and accurately test endogenous or engineered immune cell function can serve as invaluable immune oncology discovery tools.

Single cell sequencing is playing an increasingly important role in improving our understanding of the interactions between immune and cancer cells as well as how to tune the immune response for effective targeting of cancer [54,55]. For example, paired T cell receptor sequencing combined with single cell RNA sequencing enables more precise characterization of immune repertoire and immune cell state, and additional bioinformatics tools allow researchers to make inferences about immune cell state transitions and immune repertoire shaping in the context of cancer and other diseases [55,56,57]. A study by Neal et al. utilized single cell sequencing to validate a novel 3D organoid culture technique in modeling immune interactions [30]. In the study, PDOs from multiple types of cancers, including NSCLC, were generated using an air–liquid interface method. PDOs were grown in a scaffold but were exposed to air instead of being fully submerged in cell media. These PDOs exhibited expected genomic alterations and copy number variation by exome sequencing. When available, corresponding clinical sequencing data aligned closely with the PDOs genomic profile. When co-cultured with immune cells, the authors observed immune infiltration into the organoids. The study then utilized single cell sequencing, with paired B or T cell receptor sequencing and gene expression profile, to compare the immune repertoire of the organoids to the original tumor tissue. They found that their organoid model preserved the immune repertoire of the original tumor tissue over time and found that immune checkpoint treatment expanded antigen-specific tumor infiltrating lymphocytes, although the latter was only conducted using mouse organoids and not PDOs. However, these results are promising support for the use of 3D organoids, specifically those cultured using the air–liquid interface methodology, for personalized immunotherapy development.

## 7. 3D Lung Cancer Models for Neoantigen Discovery

In addition to understanding the tumor microenvironment and immune repertoire, increased understanding of the tumor antigen repertoire is vital for designing and choosing immune-based therapeutics. Tumor antigens include tumor-associated antigens (TAA), which are shared between tumor and normal tissue, and neoantigens, which arise de novo. TAAs can be more challenging to target because of concerns for off-target effects on healthy tissue and because the immune system has developed tolerance against the particular antigen, making it difficult to induce an immune response against that antigen. Neoantigens are more promising targets, but neoantigen burden varies significantly depending on the cancer’s genomic basis. Consequently, immunotherapies looking to exploit neoantigen burden have had varied efficacy. For example, the PD1 blockade checkpoint inhibitor pembrolizumab has been found to be more effective in cancer patients with defects in mismatch repair pathways and higher somatic mutation burden [58].

Aside from absolute neoantigen burden, additional factors need to be considered. Quality of neoantigens is one area that is being explored in depth with computational tools and has important implications for cell therapies and cancer vaccines [59]. Neoantigen metrics, such as ability to bind major histocompatibility molecules, affinity and avidity of the TCR-MHC complex, and neoantigen dissimilarity to the self-proteome, can be predicted and used to assess neoantigen quality as well as implications for tumor fitness likelihood of response to immune checkpoint inhibitors [60,61]. Independent of neoantigen burden and quality, HLA mutations and alterations determine whether neoantigens can be targeted by the immune system. HLA expression is disrupted in many cancers as an immune escape mechanism. For example, one study found that HLA loss occurs in 40% of early non-small cell lung cancer [62]. These losses can be broadly classified into permanent “hard lesions” or reversible “soft lesions” [63]. “Soft lesions”, such as epigenetic MHC downregulation, could potentially be reversible through treatments, such as cytokine induction, whereas “hard lesions” are permanent genomic alterations that result in non-functional MHC expression. The type of lesion influences the usefulness of neoantigen discovery.

Identification of neoantigens has traditionally been performed using mass spectrometry. However, mass spectrometry requires significant tissue input; patient biopsies and limited tissue from surgical resection limit this approach. Propagating and expanding these tissues while preserving tissue characteristics and antigens by way of 3D models help streamline the neoantigen discovery pipeline. Simultaneously, 3D models offer opportunities for manipulation and insight into both cancer pathophysiology and treatment options. For example, a study by Newey et al. utilized patient-derived organoids to analyze the immunopeptidomics of colorectal cancer [64]. The study developed a culture method that enabled significant expansion of PDOs such that there was sufficient working material for mass spectrometry. With a combination of exome sequencing, RNA sequencing, and mass spectrometry, the study identified 612 non-silent mutations across 5 patient-derived organoids but only 3 by mass spectrometry. Though the number is small, the study noted that only 0.5% of non-silent mutations result in presented neoantigens and that their technique is consistent with the lower boundary of this finding. Additionally, the study took advantage of the ability to manipulate PDOs to test the effects of IFN-gamma and MEK inhibitor treatment, demonstrating the ability to induce higher HLA and neoantigen expression. The study found that IFN-gamma treatment increased the expression of IFN-gamma inducible genes, whereas the MEK inhibitor did not alter the observed immunopeptidome.

The combination of a predictive algorithm for neoantigens coupled with a 3D model that can be manipulated and analyzed via mass spectrometry for more in-depth studies of immunopeptidomics represents yet another discovery workflow for cancer drug development in the immune oncology space.

## 8. Conclusions

In this review, we examined the current adoption of 3D models of cancer, including PDX, spheroids, and organoids in lung cancer studies. An array of technologies and bioinformatics tools have been used in the studies presented here to validate and demonstrate the potential of various 3D lung cancer models to contribute to biological understanding and therapeutic development. Genomic and RNA sequencing of 3D lung cancer models and their corresponding clinical specimens play a common role in assessing the fidelity of these models. Whole exome sequencing and gene expression profiling provide basic validation of 3D models, while single cell sequencing helps validate “next generation” 3D lung cancer models that are more complex and incorporate elements of the immune system. Additionally, the ability to conduct mass spectrometry studies using lung organoids and identify potential new antigens to target presents yet another opportunity to improve lung cancer therapy.

In certain cancer types, 3D cancer models are moving forward in full force to aid in clinical decision making and therapeutic development. The HOPE (Harnessing Organoids for Personalized thErapy) Trial, for example, aims to utilize pancreatic cancer patients’ PDOs to study drug sensitivity and personalize cancer treatment based on those studies. Drug repurposing and repositioning is also another potential avenue of cancer therapeutic advancement, and organoids have also demonstrated promise in streamlining the process [65,66].

In the era of precision medicine, preclinical cancer models should not only accurately model general features of in vivo biology but also the unique features of each patient’s cancer. The studies highlighted here demonstrate the potential research and clinical utility of 3D models of lung cancer. With additional improvements and validation, widespread adoption of these preclinical models has the potential to disrupt traditional phased drug development and significantly streamline the validation of personalized therapeutic regimens.

## Figures and Tables

**Table 1:** Overview of key technologies used to validate and study 3D cancer models as well as their therapeutic implications.

Key Technology	Use Case	Therapeutic Implications	Advantages/Disadvantages
Whole Exome Sequencing	Assess 3D cancer model’s ability to recapitulate genomic composition of tumor tissue derivative	Personalized Drug(s) Trialing and Repurposing	Enables characterization and comparison of mutation profile but does not capture gene expression levels.
RNA Sequencing	Assess 3D cancer model’s ability to recapitulate transcriptome of tumor tissue derivative	Personalized Drug(s) Trialing and Repurposing	Enables comparison of relative gene expression but does not guarantee function at the protein level.
Single Cell RNA Sequencing	Identify critical cancer cell subpopulations in 3D lung cancer models for the study of cancer stem cells and cancer evolution	Novel therapeutic regimens that target newly identified driving pathways	Enables identification of rare cell populations with functional significance. Limited throughput currently.
Single Cell RNA Sequencing with Paired TCR/BCR Sequencing	Characterize immune repertoire and immune cell states in 3D cancer and immune cell co-culture models	Development of novel immune modulating drugs and cell therapies	Enables correlation of immune cell specificity and function. Limited throughput currently.
Mass Spectrometry	Identify neoantigen burden and targets for immune-based therapies from 3D cancer models	Development of Autologous (Engineered) immune cell therapies	Enables definitive characterization of cancer cell protein expression. Requires significant sample input.

## Data Availability

No new data were created or analyzed in this study. Data sharing is not applicable to this article.
